# Impact of Acrylamide on Cellular Senescence Response and Cell Cycle Distribution via an In-vitro Study

**DOI:** 10.22037/ijpr.2021.115117.15206

**Published:** 2021

**Authors:** Elahe Mahdizade, Maryam Baeeri, Mahshid Hodjat, Mahban Rahimifard, Mona Navaei-Nigjeh, Hamed Haghi-Aminjan, Shermineh Moeini-Nodeh, Shokoufeh Hassani, Gholamreza Dehghan, Mohammad Ali Hosseinpour-Feizi, Mohammad Abdollahi

**Affiliations:** a *Department of Biology, Faculty of Natural Sciences, University of Tabriz, Tabriz, Iran. *; b *Department of Toxicology and Pharmacology, Faculty of Pharmacy, Tehran University of Medical Sciences, Tehran, Iran. *; c *Toxicology and Diseases Group, Pharmaceutical Sciences Research Center (PSRC), The Institute of Pharmaceutical Sciences (TIPS), Tehran University of Medical Sciences, Tehran, Iran. *; d *Dental Research Center, Dentistry Research Institute, Tehran University of Medical Sciences, Tehran, Iran. *; e *Pharmaceutical Sciences Research Center, Ardabil University of Medical Sciences, Ardabil, Iran.*

**Keywords:** Cellular senescence, Acrylamide, Aging, Oxidative stress, Mouse embryonic fibroblast

## Abstract

Exposure to certain environmental toxins has been shown to be associated with cellular senescence mainly through induction of oxidative stress and impact on cellular systems. Acrylamide (ACR) has raised worldwide concerns regarding the high risk of human dietary exposure to its hazardous effect. Although there is ample evidence about the carcinogenicity of ACR, limited studies have focused on its impact on cellular aging. The levels of β-galactosidase (SA-β-gal) activity, cell cycle distribution, and the expression of the senescence-associated gene and inflammatory markers were evaluated following exposure of embryonic fibroblast cells to ACR. A significant elevation in SA-β-gal activity after exposure to different concentrations of ACR was accompanied by a considerably increased level of reactive oxygen species and lipid peroxidation. ACR-treated cells showed a noticeable decline in the total antioxidant capacity and thiol molecules. Moreover, the expression of cellular senescence-related genes including p38, p53, and p21 significantly upregulated at the high concentration of 5 mM ACR. ACR also induced G0/G1 phase arrest in embryonic fibroblast cells. The current study results revealed that exposure to ACR could enhance senescence response, contributing to increased oxidative stress and cellular damage.

## Introduction

Based on the evidence, senescence has a leading role in promoting aging and age-related disorders such as chronic inflammatory diseases, cardiovascular pathologies, neurodegenerative disorders (1, 2).

Senescence is a state in which the proliferative capability of the normal somatic cells decreases after a limited proliferation leading to an irreversible cell cycle cessation. This outcome is attributed to telomere shortening and initiation of DNA damage responses (3). Besides replicative senescence, oxidative stressors, including free radicals and factors that induce inflammation, can trigger a similar senescence response (4, 5). Indeed, the role of ROS and oxidative stress have been firmly documented in the process of aging and age-related complications. Many regulatory pathways are activated following oxidative damage to macromolecules and cellular organelles, resulting in cell cycle arrest to repair the injured sites. In irreparable injury, the cells shift towards a senescence state or under extreme stress conditions towards apoptosis (6). 

Several pathways such as p53–p21 and p16INK4A-Rb are involved in controlling cellular senescence. Mitogen protein kinase (p38)/MAPK is an important signaling pathway, functions as a sensor of ROS, inflammatory stimuli, and oncogene that mediate senescence through activation of p53 (7, 8). Nuclear factor-kappa B/IKK signaling is another critical mediator pathway of the aging process activated by oxidative stress and regulates the expression of proinflammatory cytokines (9). 

There are various factors secreted by senescent cells, including different cytokines, interleukins, and growth factors that can affect surrounding cells and promote senescence response through activation of NF-kB and initiation of the p53 pathway (10). Secreted inflammatory cytokines can also promote senescence response by enhancing the senescence process in positive feedback (11). 

So far, different chemicals and environmental toxicants have been proposed for their role in the aging process (12). Acrylamide (ACR) is a highly toxic and active chemical substance used extensively in industrial activities such as paper, textiles, cosmetics, and research experiments like gel electrophoresis (13). It can also form in dietary food through Maillard reaction during the cooking process at high temperatures (14). Following human consumption of Maillard products, ACR is rapidly converted to its epoxide metabolite, glycidamide, associated with many toxic effects (15). In this regard, the reproductive toxicity, neurotoxicity, and carcinogenic properties of ACR have been confirmed in several studies (16). Mechanistically, having electrophilic properties, the carbonyl group of ACR and its metabolite could react with nucleophile sites of DNA and cysteine thiol groups of different proteins such as glutathione protamines (17). Therefore, ACR exposure reduces the cells’ glutathione content, changing the cell’s redox status and increasing oxidant products that finally lead to declining cell function and death (18, 19). Moreover, it was shown that ACR could affect the activity of oxidative stress biomarkers in rat and chicken embryos )^20^).

While ACR causes oxidative stress and irreparable oxidative injury, in severe conditions, the apoptotic pathways become activated to prevent further damage, therefore shifting the cell fate from senescence to the programmed cell death )^16^).

This research aimed to evaluate the cellular and molecular mechanism of ACR, including aging, inflammation, oxidative stress parameters, and apoptosis using the mouse embryonic fibroblast cells (NIH/3T3).

## Experimental

*Chemicals*

The β-galactosidase kit was supplied from ZellBio (GmbH, Germany). All other used compounds and reagents were obtained from Sigma-Aldrich (GmbH Munich, Germany). 

*Cell culture*

The NIH-3T3 cell line was obtained from the Pasteur Institute of Iran. The cells were cultured in DMEM (Dulbecco’s Modified Eagle Medium) containing fetal bovine serum (FBS) (10% v/v), penicillin (100 U/mL), and streptomycin sulfate (100 μg/mL). Cells were cultured in standard conditions at 37 ºC with 5% CO_2_ in a humidified atmosphere.

*The 3-(4,5-dimethylthiazol-2-yl)-2,5-diphenyl-2H-tetrazolium bromide (MTT) assay*

In this assay, MTT (3-4, 5-dimethylthiazol-2-yl-2, 5-diphenyltetrazolium bromide) tetrazolium was applied to investigate the viability of cells. In short, MTT solution (0.5 mg/mL in PBS) was added to the treated cells in 96 well-plate, and then the cells were incubated in the standard condition of cell culture. Afterward, 150 μL DMSO was added to each well, and the cells were pre-incubated at room temperature (RT) for 20 min while shaking. Finally, the absorbance of samples was evaluated at 570 nm by an ELISA (Biotech, Synergy, model Ht) reader (21).

*Cellular treatment*

After determining the viability of the NIH-3T3 cell line, five experimental groups of cells were defined, including (a) Control group; NIH-3T3 cell lines in (DMEM)-HG medium; (b) NIH-3T3+H_2_O_2_ group, the cells were exposed to H_2_O_2_ (600 μM) for 2 h in (DMEM)-HG medium as positive control; (c) NIH-3T3+ACR (1 mM); (d) NIH-3T3+ACR (2 mM); (e) NIH-3T3+ACR (5 mM) in (DMEM)-HG medium, ACR-induced cells were incubated for 24 h. The previous studies on human lung adenocarcinoma and colon adenocarcinoma cell lines showed that the IC_50_ of ACR was estimated in the range of 4.6 to 12.5 mM (22, 23). Hence, we used lower concentrations to investigate the senescence-inducing effects of ACR, including 1, 2, and 5 mM. The sub cytotoxic level of H_2_O_2_ can potentially contribute to premature senescence in NIH3T3 fibroblast cells for aging research )^24^). 

*Assessment of β-galactosidase activity*

The activity of β-galactosidase was assessed according to the manufacturer’s instructions of the β-galactosidase ELISA kit (ZellBio GmbH, Germany). The final results were measured at 450 nm using an ELISA reader.

*Determination of oxidative stress parameters*

*Lipid peroxidation (LPO) measurement*

In this reaction, lipid peroxides react with thiobarbituric acid (TBA) to form a pink color complex (Armstrong and Browne, 1994). This assay was performed based on the modified method set up in the laboratory, considering the original protocol. In brief, the REF cells were homogenized and mixed with 800 µL trichloroacetic acid, followed by centrifugation at 3000 g for 30 min. Then, the supernatant (600 µL) was mixed with 150 µL TBA (1% w/v). The resultant mixture was allowed to incubate in a boiling water bath for 15 min, followed by 400 µL of n-butanol. The ELISA reader (Synergy HT, BioTek, VT, USA) recorded the absorption of samples at 532 nm (25).

*Measurement of ROS production*

ROS level was measured using 2’,7’-dichlorofluorescein diacetate (DCFH-DA) (Hempel *et al.*, 1999). Isolated supernatants of homogenized REF cells were incubated for 30 min at 37 °C with 5 µM DCF-DA (26). In this process, the absorbance of 2’,7’-dichlorofluorescein (DCF) was determined by ELISA fluorimeter (λ ex = 488 nm, λ em = 529 nm).

*Total antioxidant power assay (TAP)*

The antioxidant potential of biological fluids/tissues was estimated using the TAP assay. In this experiment, the TAP was assessed by reduction of Fe^3+^ to Fe^2+^ and subsequent reduction of Fe^3+^ TPTZ (2, 4, 6-tris-(2-pyridyl)-s-triazine) complex to Fe^2+^ form (Benzie and Strain, 1996). The absorbance of products was recorded at 593 nm set up in the lab previously )^27^).

*Total thiol molecules (TTM)*

TTM assay was performed by a spectrophotometric technique developed by Hu (1994). The reaction of TTM with DTNB was recorded at 412 nm by the ELISA reader as set up in the lab previously (28). The supernatant of the cell extraction was mixed with 0.6 mL Tris-EDTA buffer and 40 mL 5-5’-of 10 mM dithiobis-2-nitrobenzoic acid (DTNB) in this assay. This mixture was incubated for 15 min at room temperature before being centrifuged for 10 min at 3000 g.

*Measurement of apoptosis and necrosis by flow cytometric*

According to the manufacturer’s manufacturer, this test counted viable, early, and late apoptosis and necrotic cells by Annexin V-FITC and propidium iodide (PI) kit (ApoFlowEx®FITC) instruction. Finally, cells were analyzed for the percentage of live, apoptotic and necrotic cells using a flow cytometer (Mindray, Shenzhen, China) and FlowJo software (27).

*Fluorescence microscopic analysis of cell death/apoptosis*

In this experiment, acridine orange (AO) and ethidium bromide (EB) were used for measuring viable and dead cells (apoptosis) based on the percentage of fluorescence intensity of AO/EB, using ImageJ software) 29).

*Cell cycle* *distribution using flow cytometry*

The NIH-3T3 cells were trypsinized, fixed with ice-cold ethanol 70%, and centrifuged at 10000 g for 5 min for cell cycle analysis. After washing with ice-cold PBS, the pellets were redistributed in PI having RNAse. Then, the cells were incubated at room temperature. In this method, the percentage of G0/G1, S, and G2 phases of NIH-3T3 cells were assessed by the flow cytometer as formerly described) 30).

*Expression of genes by Real-time PCR method (RT-PCR)*

To evaluate the cellular aging in NIH3T3 cells, the levels of p53, p38, p21, NF-kb, and IL6 gene expression were studied by quantitative RT-PCR. The RNA concentration extracted from our samples was evaluated using Thermo Scientific NanoDrop 2000c UV–Vis spectrophotometer. Then, cDNA synthesis was performed by iScript cDNA synthesis kit. The glyceraldehyde 3-phosphate dehydrogenase (GAPDH) primer was used as an accurate internal control. The quantitative RT-PCR was assessed by light cycler 96 system (Roch, Germany) using SYBER green master mix (Applied Biosystems, Foster City, CA, USA).

The comparative cycle threshold technique was applied to investigate the relative expression of targeted genes as set up in the lab previously) 28). The characteristics of the designed primers, including abbreviations, accession number, and sequence were shown in Table 1.

*Statistical Analysis*

The results are indicated as the mean ± standard error (SE) of the experiments. All assessments were repeated three times with five samples for each group. One-way ANOVA and Tukey tests were used for statistical and correlation analyses. StatsDirect software version 3.3.5 was used for analyzing data. The differences were considered significant if *p* < 0.05.

## Results

*Cell’s viability*

**Figure 1:**
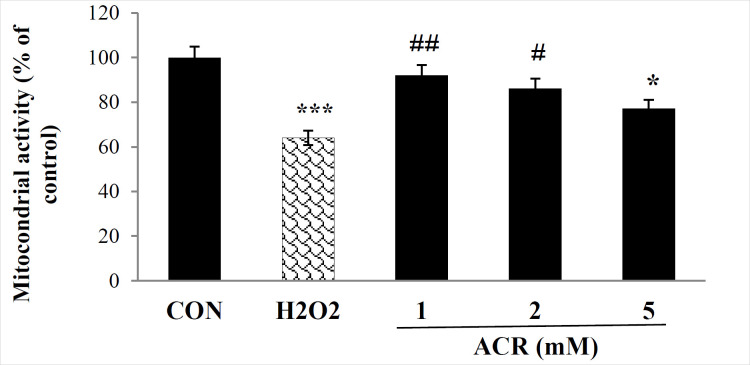
Effects of H_2_O_2_ and ACR (1, 2, and 5 mM) on mitochondrial activity in mouse fibroblast cell line NIH-3T3 by MTT test. Values are mean ± SEM. The significance of changes was reported as ^*^p < 0.05, and ^***^p < 0.001 versus the control group, ^#^*p* < 0.05, and ^##^*p* < 0.01 *versus* H_2_O_2_ group

As presented in Figure 1, the percentage of live cells significantly decreased in both 600 µM H_2_O_2_ and ACR-treated samples of 5 mM compared to the control group (*p <* 0.001, *p <* 0.05, respectively). In contrast, no significant difference was detected between control and other groups treated with various concentrations of ACR (1 and 2 mM) of ACR (*P* > 0.05).

*β-Galactosidase Activity*

**Figure 2:**
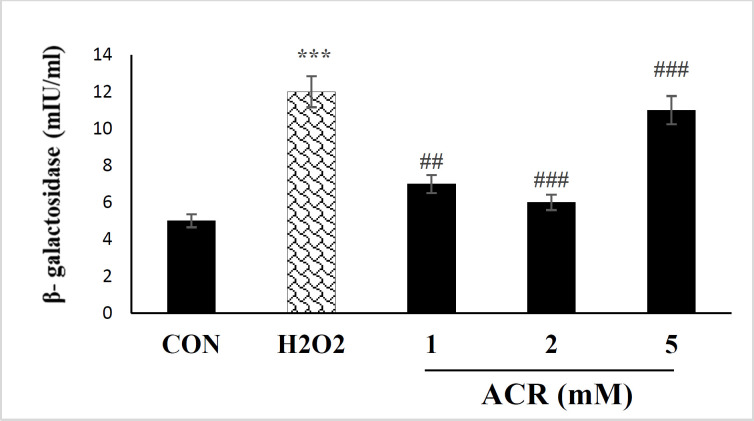
Effects of H_2_O_2 _and ACR (1, 2, and 5 mM) on β-galactosidase activity assay in mouse fibroblast cell line NIH-3T3. Values are mean ± SEM. The significance of changes was reported as ^***^p < 0.001 versus the control group, ^###^*p* < 0.001 versus H_2_O_2_ group

NIH-3T3 cells receiving ACR showed a meaningful increment in β-galactosidase activity at H_2_O_2_ and concentrations of 5 mM (*p <* 0.001) in comparison with the untreated group (Figure 2).

*Assay of oxidative stress parameters*

**Figure 3:**
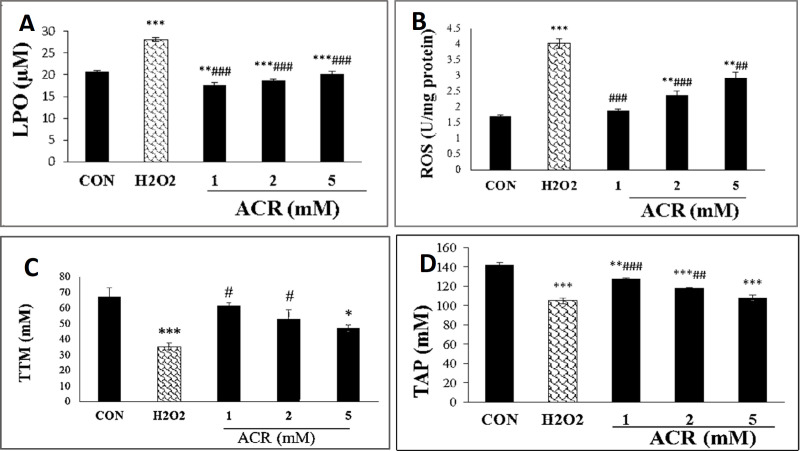
Effects of H_2_O_2_ and ACR (1, 2 and 5 mM) on oxidative stress biomarkers in mouse fibroblast cell line NIH-3T3. (A) lipid peroxidation (LPO). (B) reactive oxygen species (ROS). (C) total thiol molecules (TTM). (D) total antioxidant power (TAP). Values are mean ± SEM. The significance of changes was reported as ^*^p < 0.05, ^**^p < 0.01, and ^***^p < 0.001 versus the control group, ^#^*p* < 0.05,^ ##^*p *< 0.01, and ^###^*p* < 0.01 *versus* H_2_O_2_ group

As shown in Figure 3A, H_2_O_2_ and various concentrations of ACR (1, 2, and 5 mM) could increase the levels of LPO (*p <* 0.001). ACR at a concentration of 5 mM showed the most significant rise in LPO level compared to control (*p <* 0.001). 

Cytosolic ROS production showed a dose-dependent rise in NIH-3T3 cells, as demonstrated in Figure 3B. Also, there were no significant differences in ROS content between ACR at the 1 and 2 mM concentrations versus the untreated control group. 

The results of the TTM assay showed that exposure to 5 mM of ACR and H2O2 noticeably decreased the amount of TTM compared with the control group in NIH-3T3 cell lines (*p < *0.05*, p < *0.001, respectively). In contrast, there were no remarkable changes in the TTM level between ACR-treated groups at the concentrations of 1 and 2 mM as well as the control group (*p > *0.05) (Figure 3C).

The total antioxidant capacity was significantly decreased at 1, 2 and 5 mM of ACR in comparison with control group (*p <* 0.01, *p <* 0.001, and *p <* 0.001, respectively) (Figure 3D). 

*Flow cytometric assessment of apoptosis and necrosis*

**Figure 4:**
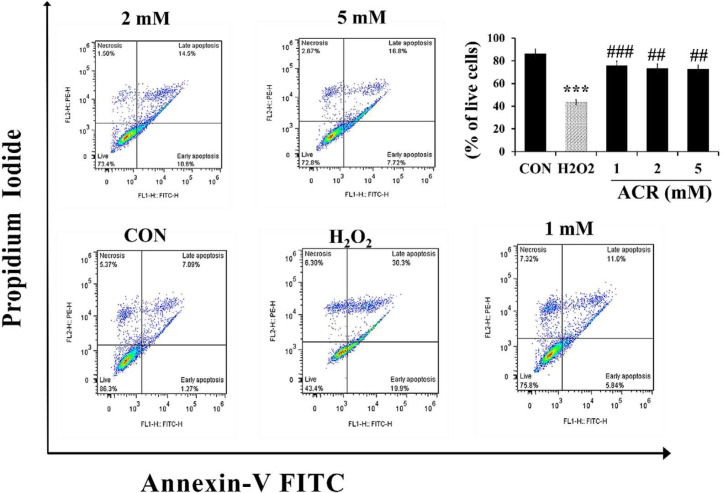
Flow cytometry evaluation of the effect of H_2_O_2_ and ACR (1, 2, and 5 mM) on mouse fibroblast cell line NIH-3T3. The lower left square shows the percentage of live cells with FITC- and PI-. The lower right square shows the percentage of early apoptotic cells with FITC + and PI-, the upper right square displays the percentage of late apoptotic cells with FITC + and PI +, and the top left square shows necrotic cells with FITC- and PI +. The significance of changes was reported as ^***^p < 0.001 *versus* the control group, ^##^*p *< 0.01, and ^###^*p* < 0.01 *versus* H_2_O_2_ group

For measuring the effect of ACR on apoptosis and necrosis, NIH-3T3 cells were exposed to various concentrations of H_2_O_2_ and ACR (1, 2, and 5 mM). As shown in Figure 4, the percentage of live cells in the H_2_O_2_ group and 5 mM ACR showed a significant reduction compared to the control group (43.4% and 72.8%, respectively). 

*Analysis of cell death/apoptosis by fluorescence microscopic*

**Figure 5:**
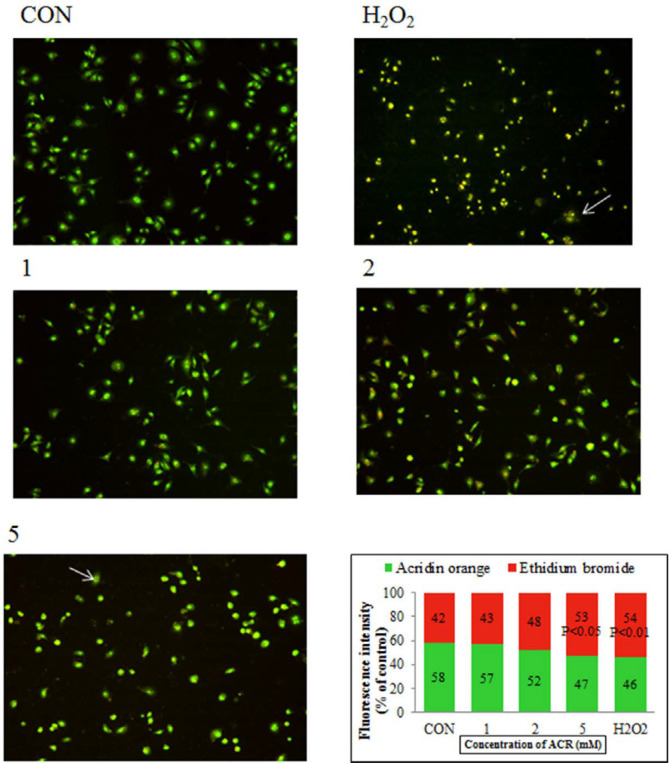
Fluorescence microscopy (magnification ×40), NIH-3T3 cells were stained with AO/EB after exposure to different concentrations of ACR and H_2_O_2_. Live cells show green fluorescence. Apoptotic cells show yellow/orange fluorescence, and dead cells show red fluorescence. The percentage of cells with green and red fluorescence was shown as a graph

Figure 5 showed AO/EB staining of NIH-3T3 cells exposed to H_2_O_2_ and different concentrations of ACR. Accordingly, 58% of the cells in the untreated group showed an integrated green color nucleus (AO). In contrast, at low doses of 1 and 2 mM ACR, 57% and 52% of cells exhibited bright green to yellow color, respectively. The cells treated with ACR (5 mM) and H_2_O_2_ had the orange-red (EB) appearance and showed nuclear disintegration (% of EB; respectively 53 and 54 *vs*. 42 in the control group). 

*Cell Cycle Analysis*

**Figure 6:**
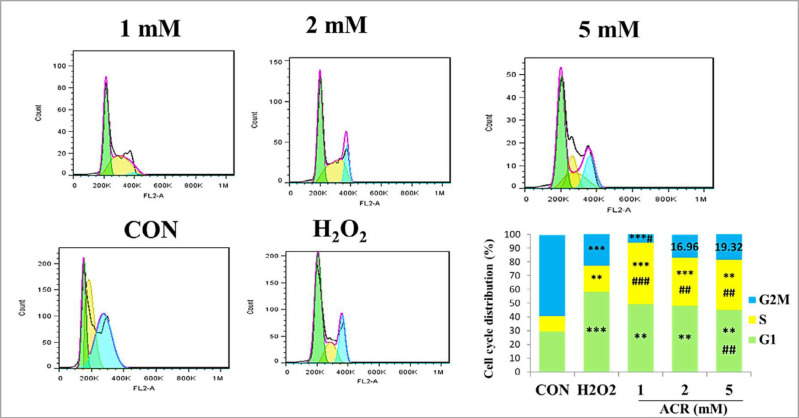
The effect of ACR (1, 2, and 5 mM) and H_2_O_2_ on mouse fibroblast cell line NIH-3T3 and cell cycle distribution assessment

Flow cytometric data of cell cycle distribution of NIH-3T3 cells showed that in the control group, 29.39% of the samples were in G1, 11.09% in S, and 59.14% in G2M (Figure 6). Exposure to different concentrations of ACR (1, 2, and 5 Mm) and H_2_O_2_-induced accumulation of cell populations in the G1 phase compared to the control group. 

*RT-PCR analysis of p53, p38, and p21*

The forward and reverse primer of p53, p38, p21, NF-κb, IL-6, and GAPDH (housekeeping) genes with accession numbers are shown in Table 1. Also, the expression changes of p53, p38, and p21 genes have been shown in Table 2. The results of RT-PCR indicated that the expressions of p53, p38, and p21 caused by the H_2_O_2_ group were more than the untreated control group (4.09-fold, 3.36-fold, and 5.76-fold, respectively; *p <* 0.001, *p <* 0.01, and *p <* 0.001, respectively). The expressions of p53, p38, and p21 have been noticeably dose-dependent upregulation in all ACR-treated groups. The results show that expression changes of p53, p38 and p21 in concentrations of 5 mM of ACR were highest compared with control group (3.69, 3.02, and 2.70; *p <* 0.01, *p <* 0.01, and *p <* 0.05) (Table 2).

*The effect of H*_2_ *O*_2_ *and ACR on the expression of NF-kb and IL-6 in NIH-3T3 cells*

The result of RT-PCR data showed that NF-kb and IL-6 gene expressions were significantly different in the H_2_O_2_ treated group compared to the control (2.61-fold and 2.38-fold; *p* < 0.01 and *p* < 0.001). As presented in Table 2, NF-kb and IL-6 were upregulated at the concentrations of 1, 2, and 5 mM of ACR in a dose-dependent manner (Table 2). The expression level of NF-kb and IL-6 indicated a significant upregulation at 5 mM of ACR (2.39-fold and 2.54-fold; *p <* 0.01 and *p <* 0.001, respectively).

*Correlation between the results*

The results in table 3 showed a noticeable positive correlation between the level of cytosolic ROS, β-galactosidase, p53, and p21 (*p <* 0.05, *p <* 0.05 and *p <* 0.01, respectively). There was also a positive correlation between β-galactosidase and p53 and proinflammatory cytokine IL6 (*p <* 0.01 and *p <* 0.05, respectively). Moreover, there was a considerable correlation (*p <* 0.01) between p53 and IL6.

## Discussion

**Figure 7:**
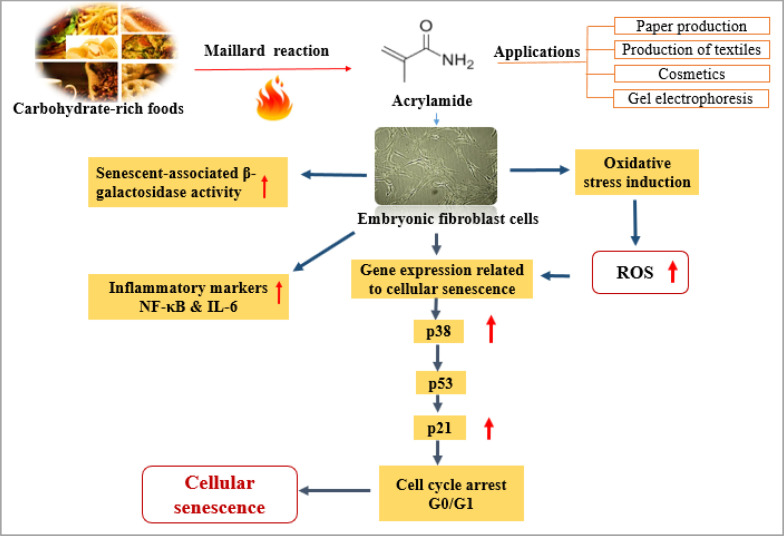
The schematic illustration of this study

In this study, we attempted to evaluate the impact of ACR on the aging of mouse embryonic fibroblasts and the underlying mechanisms. Aging is the process of gradual accumulation of deteriorative changes in organismal function and structure (31). So far, only a few studies have been shown on this aspect of ACR toxicity in cellular senescence required for further investigations. Our study showed a significant elevation in β-galactosidase level, oxidative stress, and inflammatory cytokines expression following ACR exposure. Cell cycle distribution showed that in ACR-treated fibroblast, the G0/G1 phase portion was enhanced. Along with the increased cell cycle arrest, the expression level of p38, p53, and p21 indicated a significant up-regulation at the high concentration of ACR. The schematic illustration of this study is summarized in Figure 7.

Senescence is a known condition in which cell cycle arrest occurs and is associated with the biological promotion of the aging process) 32). So far, different potential markers have been introduced for detection of senescence cell in experimental models, among which senescent-associated β-galactosidase (SA-β-gal) activity has shown to be highly specific) 33-35). SA-β-gal is the combined name for enzymes that split non-reducing β-D-galactose residues from glycoprotein, keratin sulfate, and sphingolipids in β-D-galactosidase (36). At suboptimal pH, overexpression of β-galactosidase can lead to an increase in β-galactosidase activity in senescent cells. In contrast, the activity of this enzyme in the active proliferative cells is too low (37). Our results showed that ACR induces SA-β-gal activity in embryonic fibroblast cells at different sub-toxic concentrations of ACR. The results were in line with the previous report that showed ACR could cause senescence response in macrophages and induce β-galactosidase activity through the ROS production and subsequent activation of p38 and JNK kinases )^38^). 

Oxidative stress plays a mediatory causative role in divers’ chemical-induced cellular damages) 39). DNA strand break, enzymes inactivation, and increased LPO are the significant effects of oxidative damage) 40). LPO is the underlying mechanism of many xenobiotics )^41^). Unsaturated fatty acids are the main target of LPO in which oxygenated radicals attack biological membranes and induce irreversible cellular damage. As a byproduct of LPO, malondialdehyde (MDA) can react with various macromolecules and deactivate them, resulting in cell death )^42^). Our result also suggests that ACR involves induction of ROS generation and oxidative stress markers such as LPO content, along with the subsequent decrease in the total thiol molecules and total antioxidant potential. The results were consistent with *in-vivo* studies on ACR-exposed mice and rats in which high levels of MDA and decline of antioxidant enzyme activities were reported )^20^, ^43^-^45^). The recent study confirmed the genotoxic effects of ACR, associated with elevated intracellular ROS and depletion of GSH )^46^). Consistent with these findings, using Pearson correlation, we found a significant correlation between ACR-induced-senescence response and the level of oxidative stress markers that might further implicate the significance of oxidative stress in the ACR-induced senescence response in fibroblast cells.

The high level of ROS can cause oxidative damage affecting different intracellular macromolecules and organelles) 47). Although the mechanisms of ROS-induced senescence are multifaceted and involve many signaling pathways, there are common vital regulatory factors known for their driving role in senescence response. The tumor suppressor p53 is critical for senescence cell cycle arrest and irreversible inhibition of proliferative potential, mainly involved in DNA damage-induced senescence) 1). 

P21 is the main downstream and transcriptional target of p53, which is known as a key mediator of p53-dependent cell-cycle arrest) 48). The interaction of p21 (CDKN1A) and ROS is essential for p53-mediated senescence. However, how p21 and ROS regulate each other’s expression is unclear) 49). Oxidative stress and inflammation are characteristics of senescence associated with the motivation of p38 and p53 and expression of p21 independent of p53) 50). The gene expression related to cellular senescence p53, p38, and p21 was assessed by RT-PCR to evaluate the mechanisms by which cellular aging was triggered in embryonic fibroblast cells. Our results showed that the expressions of p53, p38, and p21 genes were elevated in NIH3T3 cells when exposed to ACR as compared to the control group. Overall, the results showed that ACR significantly induced senescence in NIH3T3 cells by initiating oxidative stress and ROS production through activation of p38 and p53 pathways. 

A study of the immunotoxic effects on REF cells showed that some inflammatory mediators such as TNF-α, IL-1, IL-6, and NF-kB as a transcription factor have a determining role in inflammatory and immunologic processes (51). NF-kB is a transcriptional factor known as a proinflammatory mediator that has the central role in initiating cellular senescence, aging, and induction of stress-related gene expression and factors secreted during the senescence process such as IL-6) 52-55). The results also showed that ACR-induced senescence is associated with enhancing the expression of inflammatory factors. The inflammatory response may be due to increased oxidative stress that generation of inflammatory mediator) 56, 57). 

The apoptotic effect of ACR was confirmed in many previous studies (58, 59(. *In-vivo* studies on the nervous tissues also demonstrated the stimulatory role of ACR in ROS production and the reduction of antioxidants that leads to neurodegeneration in the rat model. Accordingly, Lakshmi et al. (58) showed that exposure to ACR increased ROS and cell apoptosis in the cerebral cortex of winter rats as determined by changes in the expression of the Bcl2 protein. Also, Liu *et al.* (18) demonstrated the mitochondrial impairment and apoptosis in an immortalized mouse microglial cell line BV2 after treating with specific concentrations of ACR. Our study using annexin V-FITC/ PI and AO/EB assay showed no significant changes in apoptosis after exposure to the low concentrations of ACR. The cell cycle analysis further confirmed the effect of ACR on senescence and apoptosis as the number of cells significantly increased in the sub-G1 phase following ACR treatment.

**Table 1:** The name, accession numbers, and primers of genes p53, p38α, p21, NF-κb, Il6, and GAPDH for RT-PCR

**Gene name**	**Gene symbol**	**Accession no.**	**Primer sequence (5′-3′)**
Tumor protein p53	*p53*	NM_001127233.1	F: CGCCGACCTATCCTTACCATR: CTTCTTCTGTACGGCGGTCT
Glyceraldehyde-3-phosphate dehydrogenase	*GAPDH*	NM_001289726.1	F: CGTGTTCCTACCCCCAATGTR: GGGAGTTGCTGTTGAAGTCG
Mitogen-activated protein kinase	*p38α*	NM_001357724.1	F: TCGGCTGACATAATTCACAGG R: GTCATCTCATCATCAGTGTGCC
Nuclear factor-kappa B	*Nfkb*	NM_008689.2	F: CCAGCTTCCGTGTTTGTTCA R: AGCTTCTGGCGTTTCCTTTG
Interleukin 6	*Interleukin 6 (Il6)*	NM_001314054.1	F: TCCAGTTGCCTTCTTGGGAC R: GTCTGTTGGGAGTGGTATCCT
KRAS proto-oncogene, GTPase (Kras)	*p21*	NM_001111099.2	F: CCTTGTCACCTCTAAGGCCA R: GGGGACCATTCCTGTCTTCA

**Table 2:** Relative mRNA expression of H_2_O_2_ and ACR (1, 2, and 5 mM) in NIH3T3

**Gene symbol**	**CON**	**H**_2_ **O**_2_	**ACR 1**	**ACR 2**	**ACR 5**
p53	1.05 ± 0.25	4.09 ± 0.47^***^	1.37 ± 0.06^##^	1.76 ± 0.39^##^	3.69 ± 0.39^**^
p38	1.05± 0.28	3.36 ± 0.041^**^	1.29±0.11^***,##^	2.94 ± 0.06^**^	3.02 ± 0.26^**^
P21	1.00 ± 0.08	5.76 ± 0.28^***^	1.41± 0.07^###^	2.30 ± 0.39^##^	2.70± 0.38^*,##^
NF-κb	1.04 ± 0.18	2.61 ± 0.21^**^	1.63 ± 0.04^#^	2.21 ± 0.32^**^	2.39 ± 0.03^**^
IL-6	1.00 ± 0.03	2.38 ± 0.09^***^	1.24 ± 0.15^##^	1.593 ± 0.17^#^	2.54 ± 0.21^***^

Results are showed as mean ± SEM. The significance of changes was reported as^ *^p < 0.05, ^**^p < 0.01, and ^***^p < 0.001 *versus* the control group,^ #^*p* < 0.05,^ ##^*p* < 0.01, and ^###^*p* < 0.01 *versus* H_2_O_2_ group.

**Table 3:** Correlations between β-galactosidase activity, levels of cytosolic ROS, p53, p21, IL6 assay

		***ROS***	***β-galactosidase***	***p53***	***IL6***	***p21***
*ROS*	Pearson Correlation	1	.902^*^	.938^*^	.866	.984^**^
	Sig. (2-tailed)		.036	.018	.057	.003
*β-galactosidase*	Pearson Correlation	.902^*^	1	.976^**^	.934^*^	.835
	Sig. (2-tailed)	.036		.004	.020	.078
*p53*	Pearson Correlation	.938^*^	.976^**^	1	.974^**^	.863
	Sig. (2-tailed)	.018	.004		.005	.059
*IL6*	Pearson Correlation	.866	.934^*^	.974^**^	1	.764
	Sig. (2-tailed)	.057	.020	.005		.133

^*^Correlation is significant at the 0.05 level (one-tailed).

^**^Correlation is significant at the 0.01 level (one-tailed).

## Conclusion

The present findings show that ACR induces premature senescence in embryonic fibroblast cells by activating the key regulators of senescence pathways; p38, p53, and p21 accompanied by increased expression of the inflammatory cytokine such as IL6 and NF-κb. The underlying mechanism is related to the formation of the free radicals and the induction of oxidative damage. Our results further support previous studies on the potential effect of ACR in the aging process. Despite the ample use of ACR, there is a lack of evidence related to the exact mechanism of cell aging caused by this compound. The main topic that needs to be evaluated is the replicative senescence biomarker, cell cycle checkpoints, cyclins and cyclin-dependent kinases (CDKs), and telomerase activity. Further *in-vivo* studies are still required to fully conclude the role of dietary ACR in aging and age-related diseases. 

## Conflict of interest

The authors declare that they have no conflict of interest.
